# COVID-19 and Parkinson’s disease: a single-center study and Mendelian randomization study

**DOI:** 10.1038/s41598-024-66197-5

**Published:** 2024-07-17

**Authors:** Jianhong Yin, Song Zhang, Qian Zheng, Zhanhui Feng

**Affiliations:** 1 Department of Neurology, The Affiliated Hospital of Guizhou Medical University, Guiyang, 550001 China; 2 Department of Neurosurgery, The Second Affiliated Hospital of Guizhou University of Traditional Chinese Medicine, Guiyang, China

**Keywords:** Parkinson’s disease, COVID-19, COVID-19 susceptibility, Motor symptoms, Mendelian randomization, Neuroscience, Diseases, Neurology

## Abstract

To investigate the association between COVID-19 and Parkinson’s disease (PD) via a single-center study and a Mendelian randomization (MR) study. A questionnaire-based survey was conducted among PD patients at a single center from December 7, 2022, to March 10, 2023. Logistic regression analysis was performed to identify the infection-related risk factors. Subsequently, bidirectional two-sample Mendelian randomization was employed to explore the association between COVID-19 and PD. In the cross-sectional analysis, it was found that the prevalence of COVID-19 infection in PD patients was 65.7%. Forty-eight (35.3%) PD patients experienced exacerbation of motor symptoms following COVID-19 infection. Long PD disease duration (≥ 10 years) (OR: 3.327, *P* = 0.045) and long time since last vaccination (> 12 m) (OR: 4.916, *P* = 0.035) were identified as significant risk factors related to infection. The MR analysis results supported that PD increases the COVID-19 susceptibility (β = 0.081, OR = 1.084, *P* = 0.006). However, the MR analysis showed that PD did not increases the COVID-19 severity and hospitalization, and no significant association of COVID-19 on PD was observed. The findings from this cross-sectional study suggest that individuals with PD may experience worsened motor symptoms following COVID-19 infection. Long disease duration (≥10 years) and long time since last vaccination (> 12 m) are identified as important risk factors for infection in these patients. Furthermore, our MR study provides evidence supporting an association between PD and COVID-19 susceptibility.

## Introduction

Coronavirus disease 2019 (COVID-19), which is caused by severe acute respiratory syndrome coronavirus 2 (SARS-CoV-2), began spreading in November 2019 and remains a global concern^1^. The government of many countries worked to reduce the spread of COVID-19 by implementing various policy interventions before the widespread spread of omicron variants. However, the rapid spread of omicron made it become the dominant variety in a short time^2^. One study demonstrated that the omicron variant showed potent immune-escape properties even in recently infected individuals, and that the variant could cause super-spread events^2^. After nearly 3 years of implementing a dynamic zero-coronavirus policy, China announced “10 new measures” to adjust the COVID-19 prevention and control strategies on December 7, 2022. Research indicated that over 70% of infections were recorded three weeks after lifting COVID-19 restrictions in Macao^3^. Another study revealed that the omicron epidemic began in Beijing in November 2022 and reached an infection rate exceeding 70%, approximately one week earlier than in Macao^4^.

Parkinson’s disease (PD) is a common neurodegenerative disease characterized by rigidity, bradykinesia, rest tremor, and postural instability^5^. A survey report revealed that the prevalence rate in China was 1.37%, with an estimated total of 3.6 million PD cases among individuals aged over 60 years^5^. The current COVID-19 pandemic caused widespread attention among neurologists and patients with PD. Previous research has observed exacerbated motor symptoms and disease progression during the COVID-19 pandemic^6^. A case of parkinsonism following COVID-19 infection has been reported^7^. And a case series of six subjects who developed PD after COVID-19 was described by Calculli A et al.^8^. Furthermore, studies have indicated that the mortality rate of COVID-19 in PD patients surpasses that of the general elderly population^9,10^. However, one study showed that available data did not yet justify a clear association between the COVID-19 pandemic and a parkinsonism wave^11^. Therefore, comprehending the intricate relationship between COVID-19 and PD is crucial for devising effective interventions to improve patient outcomes.

People with PD are recommended to receive the COVID-19 vaccine, unless they have specific contraindications^12^. A study has indicated a low vaccination rate among patients with PD^12^. However, limited information is available regarding the COVID-19 vaccination status and its protective effect on these individuals.

The present study aimed to determine the effect on patients with PD after the release of COVID-19 restrictions and analyze the infection-related risk factors of these patients, as well as evaluate the protective effect of COVID-19 vaccination on PD through a single-center study. Mendelian randomization (MR) is an effective genetic method to study the causal relationship of certain exposures to disease. Therefore, we additionally conducted a bidirectional two-sample MR analysis to explore the potential causal correlation between COVID-19 and PD.

## Methods

### Cross-sectional study design

This study had a cross-sectional design. The inclusion criteria were as follows: (1) patients diagnosed with PD according to the MDS clinical diagnosis criteria and (2) patients who maintained their medication regimen without any changes within 1 week prior to December 7, 2022. The exclusion criteria included PD patients who were unable to complete the questionnaire or lacked detailed information. Questionnaires were collected from patients who met the inclusion and exclusion criteria, who visited the outpatient and inpatient departments at the Affiliated Hospital of Guizhou Medical University between December 7, 2022, and March 10, 2023.

First, the respondents were informed that participation in the survey was greatly appreciated, that only anonymous data were collected. Second, clinical data was obtained via an interview and structured questionnaire after obtaining informed consent. The questionnaire included demographic information (age and sex), COVID-19 vaccination history (duration and type of last vaccination),PD-related clinical data (motor and non-motor symptoms), clinical features of COVID-19, as well as alterations in PD symptoms after COVID-19 infection. Third, questionnaires were completed by patients with PD themselves or with assistance from their family members if they faced difficulties in reading or writing. Trained neurologists specialized in PD administered the questionnaire. In this study, COVID-19 infection was defined as exhibiting symptoms of COVID-19 along with a positive result for COVID-19 nucleic acid or antigen between December 7, 2022, and January 7, 2023. However, symptomatic patients with negative laboratory results and asymptomatic individuals who had not been tested for COVID-19 nucleic acid or antigen were excluded from the definition of COVID-19 infection.

### Bidirectional two-sample MR study

#### Study design

**Figure 1:**
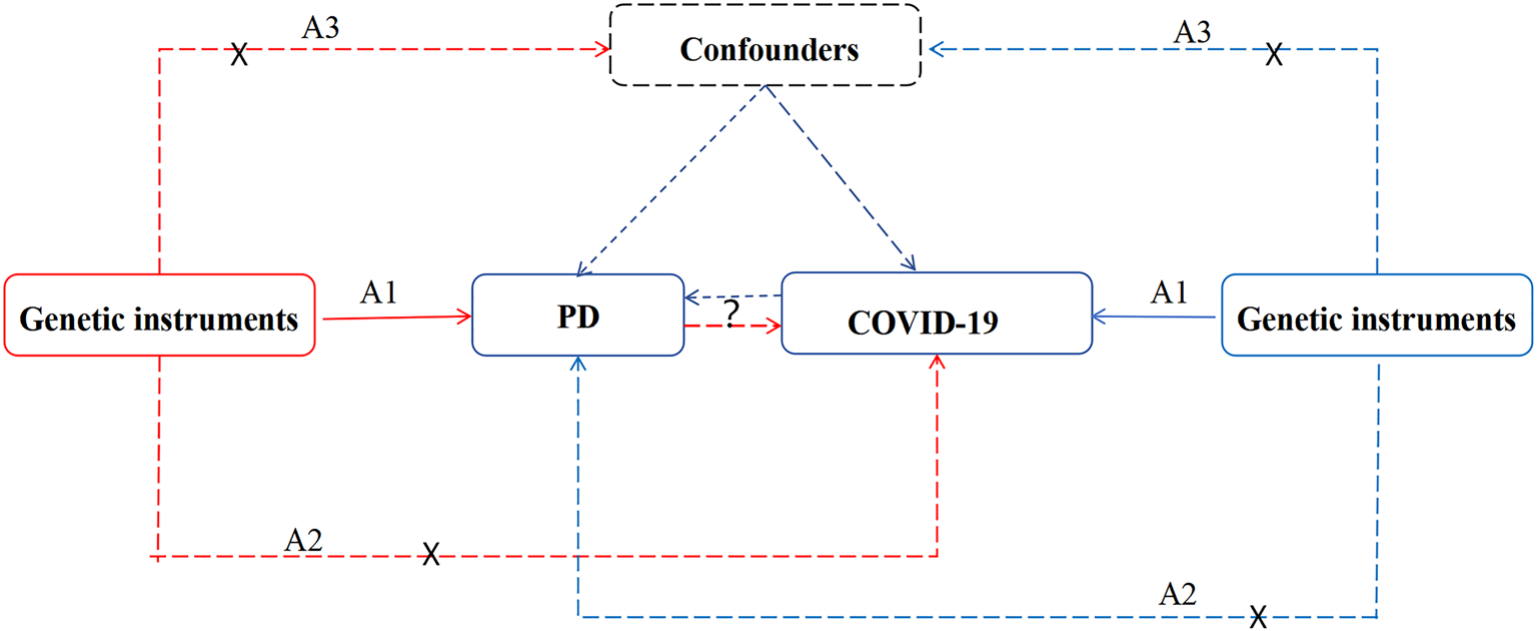
The overview of MR analysis in the study. A1: SNPs are robustly associated with exposures. A2: SNPs are not associated with confounders. A3: SNPs affect the outcome only through the exposures and not through other pathways.

In this study, we primarily used a bidirectional two-sample MR method. The MR study relies on three fundamental assumptions: firstly, there should exist a robust association between genetic variation and the exposure factor (association assumption). Secondly, the exposure factor should not possess any direct relationship with the outcome (exclusion assumption). Lastly, genetic variation should be independent of any potential confounding factors (independence assumption). The process is illustrated in Fig. 1, which presents the relevant flowchart.

#### Data sources

We used available data on COVID-19 phenotypes from the COVID-19 Host Genetics Project (RELEASE 5) based on a European population^13^. The comprehensive dataset comprises information regarding susceptibility, hospitalization, and severity of COVID-19. The susceptibility phenotype involved a comparison between COVID-19 patients and controls without COVID-19 (Ncase = 38,984, Ncontrol = 1,644,784). The hospitalization phenotype compared hospitalized COVID-19 patients with a control group that was neither hospitalized for nor infected with COVID-19 (Ncase = 9986, Ncontrol = 1,877,672). The severity phenotype compared hospitalized COVID-19 patients who died or required respiratory support with a control group without severe COVID-19 or free of COVID-19 (Ncase = 5101, Ncontrol = 1,383,241) (Table 1). In this study, COVID-19 susceptibility was diagnosed in accordance with the WHO interim guidance, or Diagnosis and Treatment Protocol for Novel Coronavirus Pneumonia (Trial Version 6), National Health Commission of the People's Republic of China.

**Table 1:** Detailed information of included GWAS datasets.

Phenotype	Population	Sample size	ncase/control	Consortium	year	GWAS ID
PD	European	482,730	33,674/449,056	IPDGC	2019	ieu-b-7
COVID-19 hospitalization	European	1,683,768	38,984/1,644,784	CHGI	2020	ebi-a-GCST011081
COVID-19 severity	European	1,887,658	9986/1,877,672	CHGI	2020	ebi-a-GCST011075
COVID-19 susceptibility	European	1,388,342	5101/1,383,241	CHGI	2020	ebi-a-GCST011073

*IPDGC* International Parkinson's Disease Genomics Consortium, *CHGI* COVID-19 Host Genetics Initiative.

The PD dataset analyzed in this study was derived from a large GWAS meta-analysis published by International Parkinson's Disease Genomics Consortium^14^. The study was also based on a European population of 482,730 participants, including 33,674 cases of PD and 449,056 healthy controls (Table 1). PD cases in this study were defined based on the UK brain bank criteria from clinic visit, or the use of PD medication or medical records of PD diagnosis by clinician, or via study participant reports of the diagnosis of PD.

### Selection of instrumental variables

The instrumental variables (single nucleotide polymorphisms, SNPs) were carefully selected based on their strong correlation with exposure (P < 5 × 10^−8^) and pruned by linkage disequilibrium (r^2^ < 0.001 and within 10,000 kb from the index variant). Outlier SNPs were identified and removed using MR Pleiotropy RESidual Sum and Outlier (MR-PRESSO)^15^. We examined whether the obtained instrumental SNPs were associated with the outcomes and the potential confounders by PhenoScanner (http://www.phenoscanner.medschl.cam.ac.uk/). The F-statistics of these SNPs were employed to evaluate the strength of each instrumental variable. The R^2^ value for each SNP was calculated using the formula: R^2^ = 2 × EAF × (1 − EAF) × β^2^^16^.

#### MR analysis and sensitivity analysis

To determine whether there is a causal association between COVID-19 and PD, we mostly employ the inverse variance weighted (IVW) method for MR analysis. Additionally, complementary methods such as MR-Egger, weighted median, simple mode, and weighted mode were utilized in conjunction with IVW. The Cochran’s Q test of the IVW approach and leave-one-out analysis were used to investigate the degree of heterogeneity. The MR-Egger intercept test and MR-PRESSO global test were employed to evaluate the horizontal pleiotropy.

### Statistical analysis

Statistical analyses were performed using SPSS statistical software for Windows version 24.0 (SPSS, Chicago, IL, USA) for cross-sectional study. The independent sample t-test was employed to analyze continuous variables with normal distributions, including general data and clinical basic information. Chi-square test and Fisher exact test were used to compare categorical variables. Logistic regression analysis was performed to analyze the infection-related risk factors. A significance level of *P* < 0.05 indicated statistical significance.

For MR analysis, all analyses were carried out in R (version 4.3.0) utilizing the TwoSample MR and MR-PRESSO packages^15–17^. *P* < 0.05 was considered as nominally significant and *P* < 0.05/6 (0.008) was considered as significant after correcting by Bonferroni measures.

### Ethics approval

The protocol for the research project has been approved by a suitably constituted Ethics Committee of the institution within which the work was undertaken and that it conforms to the provisions of the Declaration of Helsinki (as revised in Fortaleza, Brazil, October 2013).

### Approval of the research protocol

The study was conducted in accordance with the Declaration of Helsinki and was approved by the Affiliated Hospital of Guizhou Medical University.

### Informed consent

All participants provided informed consent before enrollment.

## Results

### Cross-sectional study

#### Characteristics of patients with PD

In the period of study, a total of 258 patients with PD were admitted to our hospital. Among them, 209 patients fulfilled the inclusion and exclusion criteria and were invited to participate in this study. Two patients were excluded due to insufficient information provided. Finally, a total of 207 PD patients participated in this study. The baseline characteristics and clinical features of both PD and COVID-19 are presented in Table 2.

**Table 2:** The basic characteristics of participants (*n* = 207).

Characteristics	COVID-19 infected group (*n* = 136)	Uninfected group (*n* = 71)	t/χ^2^	*P* value
Age, mean (SD), years	66.83 (11.26)	68.58 (9.75)	−1.108	0.269*
Females, no. (%)	66 (48.5)	35 (49.3)	0.011	0.917^**†**^
Course of PD				
< 3 years, no. (%)	47 (34.6)	27 (38.0)	2.596	0.458^**†**^
3–4 years, no. (%)	31 (22.8)	21 (29.6)		
5–9 years, no. (%)	31 (22.8)	14 (19.7)		
≥ 10 years, no. (%)	27 (19.9)	9 (12.7)		
Motor symptoms				
Rest tremor, no. (%)	107 (78.7)	56 (78.9)	0.001	0.974^**†**^
Bradykinesia, no. (%)	135 (99.3)	71 (100)	0.525	0.469^**†**^
Rigidity, no. (%)	122 (89.7)	68 (95.8)	2.279	0.131^†^
Posture instability, no. (%)	88 (64.7)	49 (69.0)	0.387	0.534^**†**^
Time since last COVID-19 vaccination				
< 3 months, no. (%)	6 (5.5)	6 (10.7)	19.998	** < 0.001**^**†**^
4–6 months, no. (%)	15 (13.8)	22 (39.3)		
7–12 months, no. (%)	45 (41.3)	20 (35.7)		
> 12 months, no. (%)	43 (39.4)	8 (14.3)		
Vaccination type				
Without vaccination, no. (%)	27 (19.9)	14 (19.7)	2.395	0.663^†^
Vero, no. (%)	65 (47.8)	40 (56.3)		
Cho, no. (%)	31 (22.8)	12 (16.9)		
Adenovirus, no. (%)	5 (3.7)	3 (4.2)		
Unknow, no. (%)	8 (5.9)	2 (2.8)		

*Independent sample t-test was used for the analyses of continuous variables and had normal distributions.

†Chi-square test was used to compare categorical variables.

Significant values are in bold.

A total of 106 male and 101 female participants were enrolled in this study. The mean age of the participants was 67.76 years (SD ± 9.71, range 39–88), with a mean PD duration of 5.24 years. Among the PD patients, a total of 136 individuals were infected with COVID-19, resulting in an infection rate of 65.7%. The mean ages of the patients infected or uninfected with COVID-19 were 66.83 (SD ± 11.26, range 39–84) and 68.58 (SD ± 9.75, range 46–88) years, respectively (*P* = 0.269). No significant differences were observed between the COVID-19-infected and uninfected groups regarding sex (*P* = 0.917), courses of the PD (*P* = 0.458), vaccination status (*P* = 0.663), rest tremor (*P* = 0.974), bradykinesia (*P* = 0.469), rigidity (*P* = 0.131), or posture instability (*P* = 0.534). However, the result revealed a significant difference between the COVID-19-infected and uninfected groups in terms of time since last COVID-19 vaccination (*P* < 0.01).

#### Clinical features of COVID-19 in patients with PD

**Figure 2:**
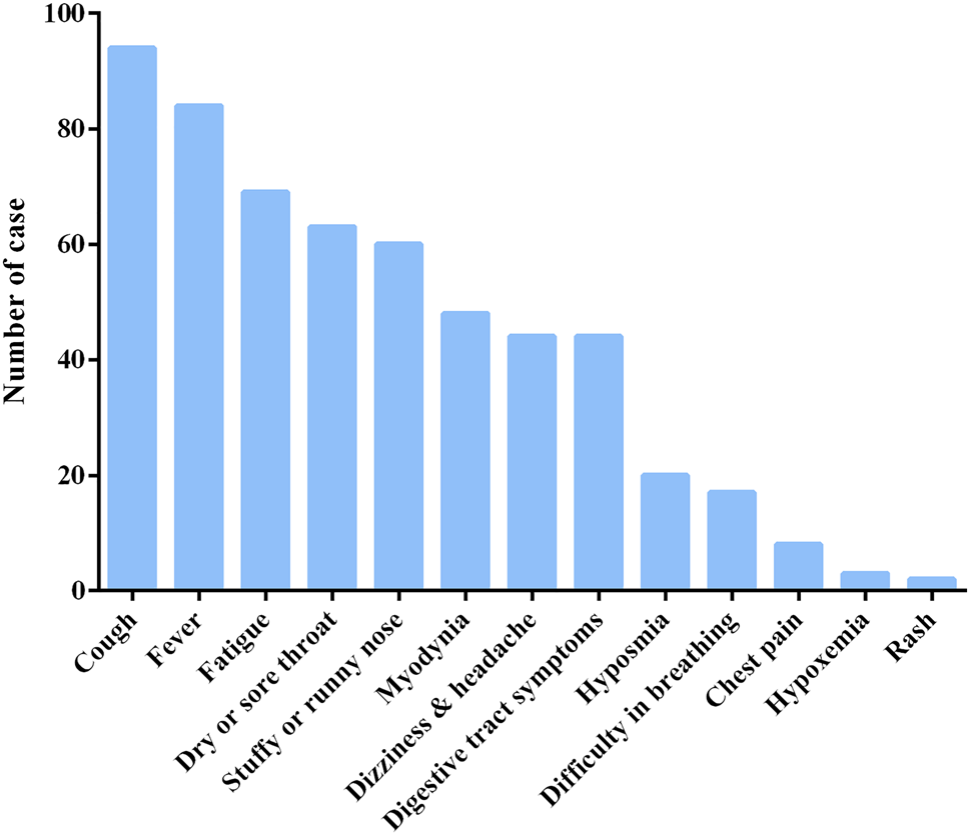
Clinical manifestations of PD patients after COVID-19 infection.

In this study, a total of 136 individuals were included, consisting of 70 males and 66 females who tested positive for COVID-19. The observed symptoms among these patients encompassed cough (*N* = 94), fever (*N* = 84), fatigue (*N* = 69), dry or sore throat (*N* = 63), stuffy or runny nose (*N* = 60), myodynia (*N* = 48), dizziness and headache (*N* = 44), digestive tract symptoms (*N* = 44), hyposmia (*N* = 20), difficulty breathing (*N* = 17), chest pain (*N* = 8), hypoxemia (*N* = 3), and rash (*N* = 2) (Fig. 2). Among them, approximately half of the cases reported a duration of COVID-19 symptoms as 1 week (50.74%), followed by 2 weeks in around one-third (29.41%), while over 2 weeks accounted for nearly one-fifth (19.85%).

**Figure 3:**
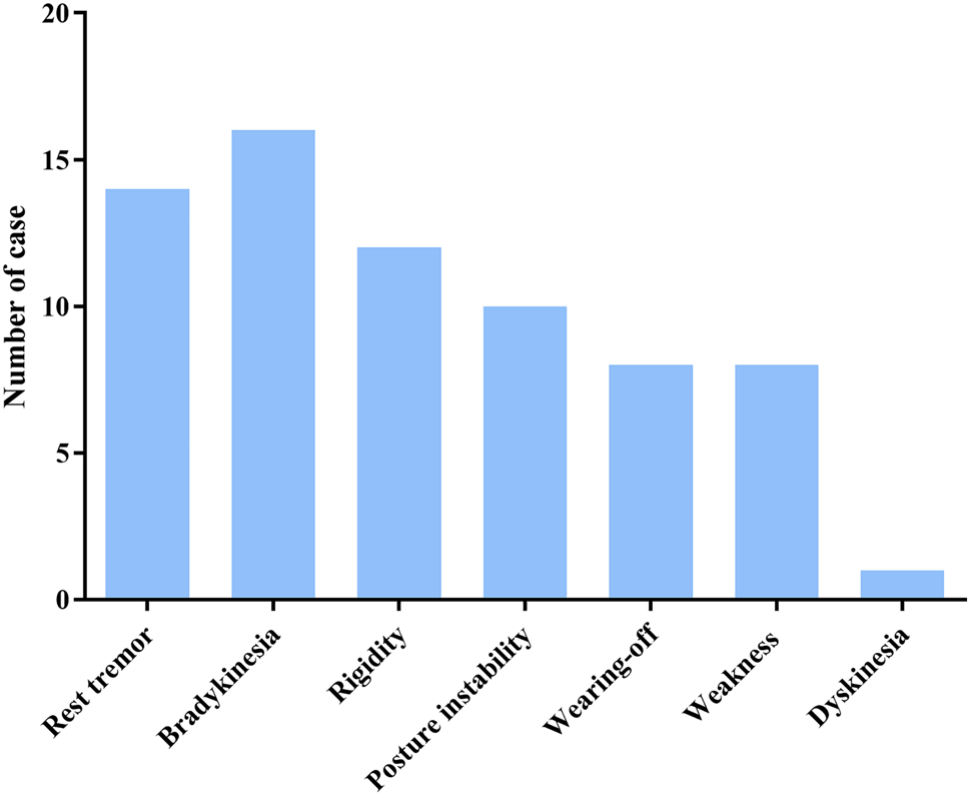
Manifestations of worsened PD motor symptoms after COVID-19 infection.

Forty-eight patients experienced a worsening of motor symptoms, resulting in an exacerbation rate of 35.3%. The deteriorated motor symptoms included rest tremor (*N* = 14), bradykinesia (*N* = 16), rigidity (*N* = 12), posture instability (*N* = 10), wearing-off (*N* = 8), weakness (*N* = 8), and dyskinesia (*N* = 1) (Fig. 3). During the questionnaire period, it remained uncertain whether the worsening of PD symptoms was transient or permanent, necessitating further monitoring of symptom progression in these patients at subsequent stages. Sixteen (11.8%) patients with PD were admitted to hospitals due to COVID-19 infection, and one fatality occurred as a result of respiratory failure. More than half of the hospitalized patients had an average hospitalization duration of one week.

#### Infection-related risk factors and protective effect of COVID-19 vaccine

A total of 166 patients with PD were vaccinated against COVID-19, with a vaccination rate of 80.2%. Logistic regression analysis was conducted to investigate the infection-related risk factors in these patients, considering age, sex, course of PD, time since last vaccination, and type of the last dose of vaccination as independent variables. The findings revealed that long disease duration (≥ 10 years) (OR = 3.327, 95% CI 1.029–10.755) and long time since last vaccination (> 12 m) (OR = 4.916, 95% CI 1.116–21.650) were identified as significant risk factors (Table 3). Notably, individuals who received their COVID-19 vaccine within three months exhibited a lower infection rate compared to those vaccinated after three months (50% vs. 67.3%). These results suggest that the COVID-19 vaccine provides short-term protection for patients with PD.

**Table 3:** Multivariate logistic regression analysis for screening the COVID-19 infection predictors in PD patients.

Index	OR	95% CI	*P* value
Age	0.977	0.937–1.020	0.291
Sex (ref: male)			
Female	1.613	0.782–3.329	0.196
Course of PD (ref: < 3 years)			
3–4 years	1.189	0.486–2.906	0.704
5–9 years	2.444	0.829–7.204	0.105
≥ 10 years	3.327	1.029–10.755	0.045*
Time since last COVID-19 vaccination (ref: < 3 months)			
4–6 months	0.531	0.127–2.218	0.385
7–12 months	2.113	0.558–8.008	0.271
> 12 months	4.916	1.116–21.650	0.035*
Vaccination type (ref: without vaccination)			
Vero	1.399	0.569–3.435	0.464
Cho	1.557	0.312–7.769	0.589
Adenovirus	1.497	0.251–8.942	0.658
Unknow	2.452	–	0.583

*Ref* reference, *Vero* inactivated COVID-19 vaccine, *Cho* recombinant COVID-19 vaccine, *adenovirus* adenovirus vaccine for COVID-19.

*P value is significant.

### MR analysis results

To investigate the causal linkage of COVID-19 on PD, three COVID-19 traits (hospitalization, severity, and susceptibility) were used as exposures. PD data from the IEU database served as the outcome variable. For each trait, a set of 4, 8, and 5 instrumental SNPs were respectively included. On the other hand, to investigate the causal linkage of PD on COVID-19 outcomes, we selected sets of 8, 21, and 12 instrumental SNPs for genetically predicting COVID-19 hospitalization, severity, and susceptibility after excluding palindromic SNPs as well as potential pleiotropy or outliers. These selected SNPs were strongly correlated (*P* < 5E−8) (Supplemental Tables 1 and 2) and independent (R^2^ < 0.001) (Supplemental Table 3) for exposure. All F-statistics were greater than 10 (Supplemental Table 3).

**Figure 4:**
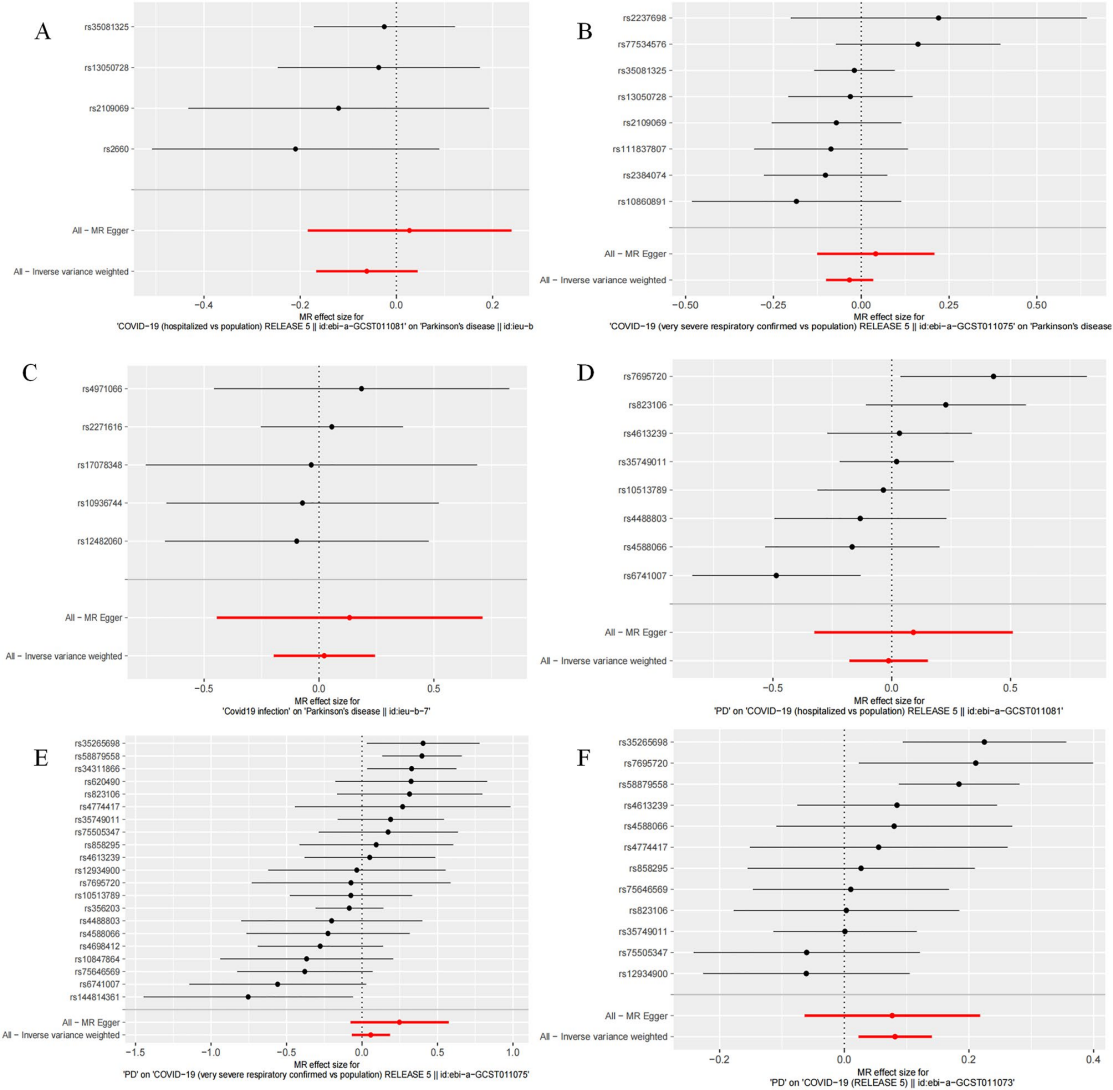
MR estimates for the causality of the association between COVID-19 and PD. Panels (**A–C**) respectively indicate the causal estimates of COVID-19 hospitalization, severity, and susceptibility on PD. Panels (**D–F**) respectively indicate the causal estimates of PD on COVID-19 hospitalization, severity, and susceptibility.

The study findings did not reveal any statistically significant impact of COVID-19 on the increased risk of PD (Hospitalization: OR = 0.979, 95% CI: 0.847–1.044, *P* = 0.249; Severity: OR = 0.967, 95% CI: 0.905–1.033, *P* = 0.322; Susceptibility: OR = 1.023, 95% CI: 0.821–1.274, *P* = 0.840) (Table 4, Fig. 4) .

**Table 4:** Mendelian randomization estimates for associations between COVID-19 and PD.

Exposure	Outcome	Beta	95% CI	*P-*IVW	*P-*heterogeneity	*P-*intercept	*P-*PRESSO
COVID-19 hospitalization	PD	−0.062	0.847–1.044	0.249	0.711	0.443	0.738
COVID-19 severity		−0.034	0.905–1.033	0.322	0.529	0.374	0.587
COVID-19 susceptibility		0.023	0.821–1.274	0.840	0.965	0.713	0.966
PD	COVID-19 hospitalization	−0.014	0.837–1.162	0.871	0.035	0.608	0.056
	COVID-19 severity	0.058	0.935–1.201	0.362	0.008	0.229	0.008
	COVID-19 susceptibility	0.081	1.023–1.149	0.006*	0.048	0.945	0.058

*CI* confidence interval, *IVW* inverse variance-weighted, *P-heterogeneity* P-value for heterogeneity using Cochran’s Q test, *P-intercept* P-value for MR-Egger intercept, *P-PRESSO* P-value for MR-PRESSO global test.

*P value is significant.

**Figure 5:**
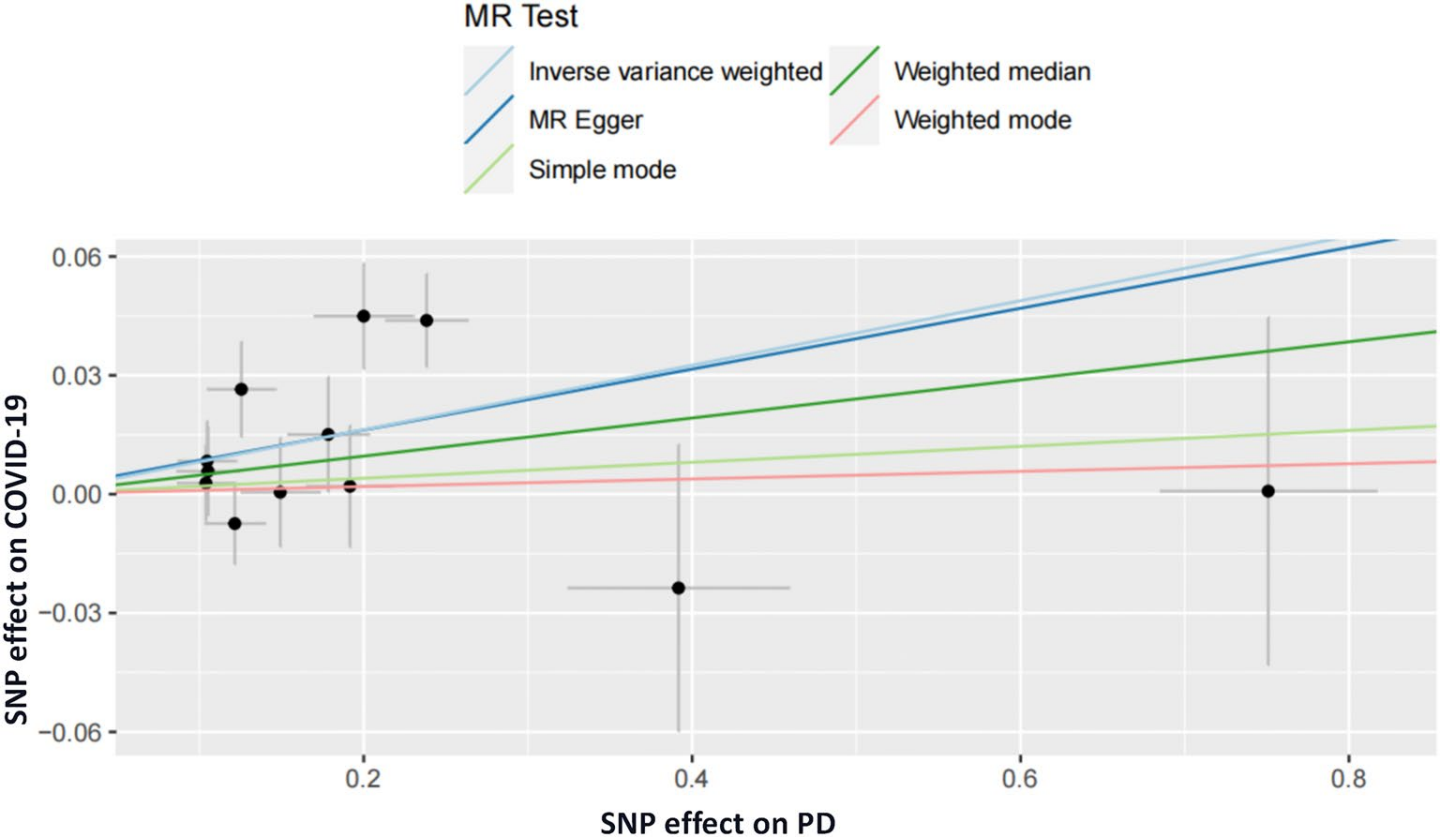
Scatter plot of the MR estimate for the effect of PD on the risk of COVID-19 susceptibility.

We also observed no statistically significant effect of PD on the increased risk of COVID-19 hospitalization and severity (hospitalization: OR = 0.987, 95% CI 0.837–1.162, *P* = 0.871; severity: OR = 1.060, 95% CI 0.935–1.201,* P* = 0.362). However, the results of the IVW method provided evidence supporting an association between PD and increased susceptibility to COVID-19 (OR = 1.084, 95% CI 1.023–1.149, *P* = 0.006) (Table 4, Figs. 4 and 5).

The existence of gene pleiotropy was tested using MR-Egger regression analysis, and all intercept terms were found to be close to zero (*P* > 0.05). The MR-PRESSO method was also employed and yielded consistent results with the MR-Egger regression. Although the* P*-values of the MR-PRESSO analysis was less than 0.05 in COVID-19 severity, horizontal pleiotropy did not present in the result of MR-PRESSO destruction test (*P* > 0.05). Furthermore, Cochran's statistical test revealed no significant heterogeneity effect (Q-value > 0.05) (Table 4).

## Discussion

The rapid spread of the COVID-19 pandemic poses particular challenges for the management of patients with PD^18^. One study showed that mortality and hospitalization rates among elderly individuals with PD did not significantly differ from those of the general population^18^. However, another study revealed a higher COVID-19 mortality rate among PD patients compared to other hospitalized individuals^19^. Hence, the association between COVID-19 and PD remains unclear. Furthermore, on December 7, 2022, China introduced "10 new measures" to adjust strategies for preventing and controlling COVID-19. Therefore, further investigation is required to identify the relationship between COVID-19 and PD and develop future intervention measures.

The findings of one study revealed that within 3 weeks after the relaxation of COVID-19 restrictions in Macao, China, the omicron variant epidemic exhibited a staggering infection rate of 75%^3^. Additionally, another study indicated that the omicron epidemic reached an infection rate exceeding 70%, approximately one week earlier than observed in Macao^3^.These studies provide suggestive evidence regarding post-restriction COVID-19 infections. Furthermore, our present study demonstrated the rapid transmission of the omicron variant among patients with PD, with an infection rate of 65.7% within a month following the release of COVID-19 restrictions in China.

One study revealed that over 60 percent of individuals infected with COVID-19 exhibited symptoms such as fever, dry or sore throat, nasal congestion and runny nose, fatigue, headaches, or muscle aches^3^. Similarly, the predominant COVID-19 symptoms observed in patients with PD in this study included coughing, fever, fatigue, dry or sore throat, nasal congestion or runny nose, myalgia, dizziness and headache along with gastrointestinal symptoms. More than half of PD patients experienced COVID-19 symptoms within one week.

The effect of COVID-19 on patients with PD is multifaceted. Several studies have indicated that COVID-19 infection may exacerbate both motor and non-motor symptoms of PD, or give rise to previously unobserved symptoms^20,21^. A study presented two patients with PD who underwent subthalamic deep brain stimulation and experienced rapid deterioration in their PD symptoms subsequent to contracting COVID-19^22^. In this current study, over one-third of the patients with PD exhibited aggravated motor symptoms, with bradykinesia being the most prominent. However, it remained unclear during the questionnaire period whether these worsened PD symptoms were transient or permanent, necessitating further monitoring of symptom evolution in these patients at a later stage. Studies have suggested that COVID-19 infection may aggravate PD symptoms through potential mechanisms such as hematologic pathways or axonal transport via olfactory neuroepithelium, leading to inflammation, immunologically mediated mitochondrial injury, and neuronal oxidative stress^8,23^. One study reported an increased risk ratio (RR) of developing PD 6 and 12 months after a positive COVID-19 test compared to individuals who tested negative for COVID-19^24^. However, it is important to note that any intercurrent illness (e.g. flu, urinary infection, pneumonia) can temporarily exacerbate PD symptoms. Further research is needed to compare the exacerbation of PD symptoms between COVID-19 and other concurrent illnesses. Therefore, we performed a two-sample MR analysis to determine the association between COVID-19 and PD. However, we found no statistically significant effect of COVID-19 susceptibility, hospitalization and severity on the increased risk of PD at the genetic level. Nevertheless, it is important to acknowledge that this analysis may be constrained by data limitations, necessitating further cohort studies to explore this relationship.

Vaccination is a highly effective measure for preventing severe illness caused by COVID-19 and reducing the transmission of infection. A study revealed that the COVID-19 vaccination rate among patents with PD was 54.0%^12^. However, in our present study, the COVID-19 vaccination rate was found to be higher at 80.2% compared to the previous study. Furthermore, our results indicated that the long time since last vaccination (> 12 m) was one of the infection-related risk factors of patients with PD. Additionally, it was observed that the COVID-19 vaccine provided short-term protection for individuals with PD. Therefore, we recommend COVID-19 vaccination for PD patients unless there are specific contraindications.

There was no significant difference in the risk of COVID-19 infection between PD patients and healthy controls during the COVID-19 pandemic according to one study^18^, indicating that PD is not a contributing factor to the susceptibility of COVID-19. However, another study showed that PD patients possess a higher rate of COVID-19 mortality^19^. The findings of the present study showed that long disease duration (≥ 10 years) was another infection-related risk factor. Therefore, we further determine the association between PD and COVID-19, and our MR study provided evidence supporting a significant association between PD and an elevated susceptibility to COVID-19. However, we did not find any statistically significant effect of PD on the increased risk of hospitalization or severity related to COVID-19 at the genetic level.

Due to the change in China’s current COVID-19 management policy, understanding the effect on patients with PD after the release of COVID-19 restrictions and taking timely preventive measures in the future, such as administrating booster vaccine and stockpiling antiviral drugs, could reduce the morbidity and mortality of these patients in the future epidemic wave.

There were several limitations to our study. Firstly, COVID-19 infection in our study was defined as the presence of respiratory symptoms along with positive nucleic acid tests or antigen detection. However, it is important to acknowledge that asymptomatic individuals who did not undergo nucleic acid or antigen testing were not included in our study cohort, potentially leading to an underestimation of the true infection rate. Secondly, the analysis did not differentiate PD disease severity separately, which may result in overlooking distinctions between PD disease severity and COVID-19. Thirdly, it remains unclear whether the worsening of PD symptoms was transient or permanent. Lastly, it should be noted that this study did not investigate the association between previous COVID-19 infections and PD within different timeframes due to data limitations. Therefore, additional cohort studies are required to investigate this relationship.

## Conclusions

More than one-third of patients with PD exhibits exacerbated motor symptoms, with bradykinesia being the most prominent. The findings from this cross-sectional study suggest that COVID-19 infection contributes to the deterioration of motor symptoms in PD patients. Notably, long disease duration (≥ 10 years) and long time since last vaccination (> 12 m) are identified as risk factors associated with infection. Furthermore, the MR study supports the association between PD and a higher susceptibility to COVID-19. In addition, PD patients are encouraged to be receive booster vaccine because of the short-term protective effect of COVID-19 vaccine on them.

### Supplementary Information

Supplementary Tables.

## Data Availability

The datasets used and/or analysed during the current study available from the corresponding author on reasonable request.
